# An experimental perspective on nanoparticle electrochemistry

**DOI:** 10.1039/d4cp00889h

**Published:** 2024-06-18

**Authors:** Esperanza Sedano Varo, Rikke Egeberg Tankard, Julius Lucas Needham, Esteban Gioria, Filippo Romeggio, Ib Chorkendorff, Christian Danvad Damsgaard, Jakob Kibsgaard

**Affiliations:** a Department of Physics, Technical University of Denmark 2800 Kongens Lyngby Denmark jkib@fysik.dtu.dk; b Center for Visualizing Catalytic Processes (VISION), Department of Physics, Technical University of Denmark 2800 Kongens Lyngby Denmark; c National Centre for Nano Fabrication and Characterization, Technical University of Denmark 2800 Kongens Lyngby Denmark

## Abstract

While model studies with small nanoparticles offer a bridge between applied experiments and theoretical calculations, the intricacies of working with well-defined nanoparticles in electrochemistry pose challenges for experimental researchers. This perspective dives into nanoparticle electrochemistry, provides experimental insights to uncover their intrinsic catalytic activity and draws conclusions about the effects of altering their size, composition, or loading. Our goal is to help uncover unexpected contamination sources and establish a robust experimental methodology, which eliminates external parameters that can overshadow the intrinsic activity of the nanoparticles. Additionally, we explore the experimental difficulties that can be encountered, such as stability issues, and offer strategies to mitigate their impact. From support preparation to electrocatalytic tests, we guide the reader through the entire process, shedding light on potential challenges and crucial experimental details when working with these complex systems.

## Abstract 2

While model studies with small nanoparticles offer a bridge between applied experiments and theoretical calculations, the intricacies of working with well-defined nanoparticles in electrochemistry pose challenges for experimental researchers.

## Graphical abstract

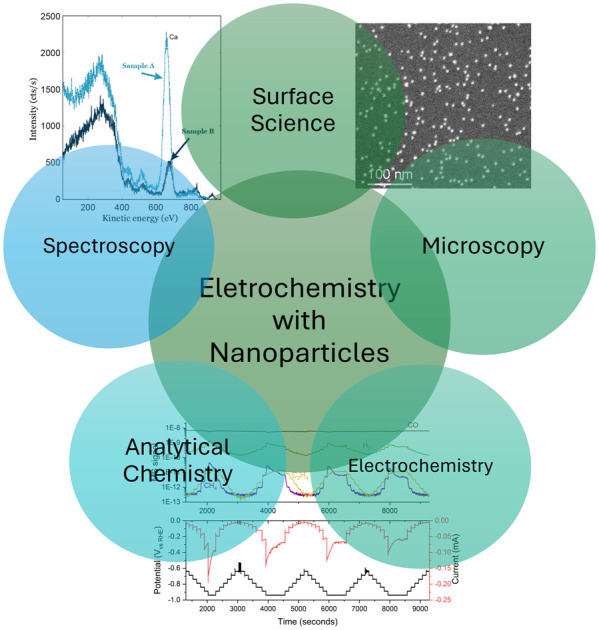

## Introduction

As scientific research methods advance to address global energy challenges, sub-fields and individual experimental methods are becoming increasingly specialized. The techniques required in today's scientific research are highly complex, forcing researchers to often specialize in sub-segments of the overall research objective. Consequently, research projects must embrace a multidisciplinary approach, where various specialists collaborate to gain the most accurate understanding of the subject under investigation. Research progresses through the intersections of disciplines, and within these intersections, understanding the intrinsic electrochemical activity of nanoparticles stands out as an example of a complex one. This field combines aspects of chemistry, physics, engineering, surface science, and nanoscience, requiring the establishment of a methodological approach to facilitate this interdisciplinary collaboration.

Understanding the intrinsic activity of nanoparticles is crucial for optimizing their overall performance. However, experimentally, this quest encounters challenges due to the difficulty in eliminating the influence from external factors such as contamination, particle agglomeration, and support interactions. To surmount these challenges and reduce the impact of external parameters, it is essential to work with small, controlled nanoparticle loadings on well-defined supports. In relation to this, the uncertainties on the sizes and loadings of nanoparticles produced, as well as influence from contamination in the support on the activity measurements, must be carefully investigated and understood. Once these experimental parameters are firmly established, and the experiments prove robust, meaningful conclusions about factors like nanoparticle size, shape, composition or loading effects on their performance in each reaction can be drawn.

Flat surfaces as a support offer several advantages for nanoparticle studies, including ease of characterization and the ability to control nanoparticle dispersion. Nevertheless, working with flat surfaces introduces challenges, particularly when only covering a small fraction of the electrode's surface area with the catalytic nanoparticles. The extensive available surface area of flat supports (relative to the catalyst's area) can result in contamination from the support material, if this support contains metal impurities that are also active under the same reaction conditions, potentially masking the intrinsic activity of the nanoparticles. Additionally, nanoparticles on flat surfaces may interact with each other, leading to aggregation or sintering, which can further alter their activity.^1^

Reproducibility is another critical aspect of nanoparticle studies. To ensure reliable and meaningful results, it is essential to employ reproducible synthesis, characterization and activity test methods. This requires careful control over variables such as reaction conditions, nanoparticle size distribution, and coverage of the electrode's surface. By carefully considering these factors, researchers can gain a deeper understanding of the intrinsic activity of nanoparticles and pave the way for the development of advanced nanomaterials with tailored properties.

**Fig. 1:**
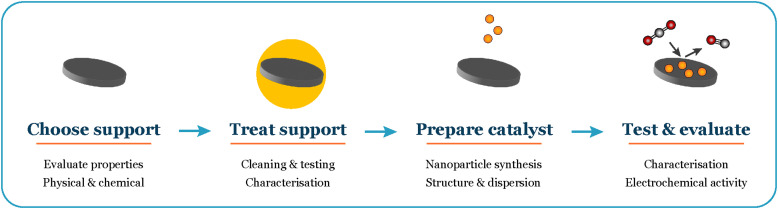
Overall workflow when working with supported nanoparticles in electrochemistry.

In this perspective, we share insights gained from our experiments with extremely low loadings (150–500 ng cm^−2^) of size-selected nanoparticles for CO and CO_2_ electroreduction. We present our unique experimental approach and vision for navigating these intricate systems (Fig. 1). Our objective is to offer a thorough guide for experimentalists working with well-defined systems, empowering them to achieve reliable results in electrochemistry.

## Substrate choice and preparation – cleaning and pre-treatment before UHV

**Fig. 2:**
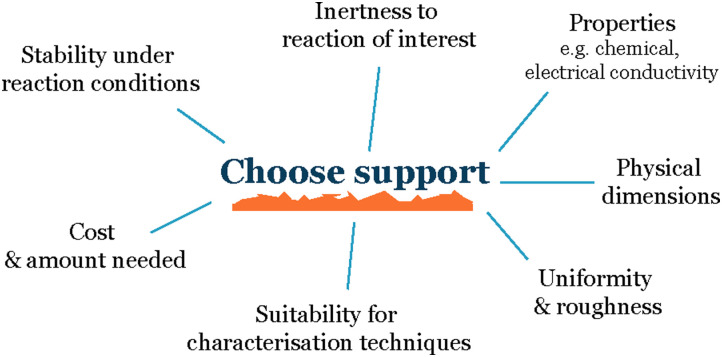
Parameters to consider when choosing a support.

When working with nanoparticles and small clusters in electrochemistry, the first choice that needs to be made is the electrode material onto which we deposit our electrocatalysts (Fig. 2). The main characteristics of this support material must be:

Inert to the electrochemical environment – the support material should not react with the electrolyte, reactants, or products, as this can interfere with the electrochemical reaction of interest and make it difficult to isolate the activity of the nanoparticles.

Stable over time – it should not degrade, and the support properties such as roughness or composition should not change over time, as this can also affect the activity of the nanoparticles.

Conductive – the substrate should be conductive to allow for the flow of electrons between the nanoparticles and the electrode.

Uniform – the support material should have a uniform topography to avoid uneven nanoparticle distribution due to differences in the roughness of the support. This is important because uneven nanoparticle distribution can lead to variations in the interparticle distance and promote sintering, which makes it difficult to establish a reproducible system.

Stabilize the nanoparticles on the surface – to the greatest extent possible, the substrate should be able to stabilize the nanoparticles on the surface to prevent them from aggregating or sintering. This is important because aggregation and sintering can reduce the active surface area of the nanoparticles and make the electrode less active over time.

The choice of support material will depend on the specific reaction and system being studied. In general, graphite, in the form of glassy carbon or highly oriented pyrolytic graphite (HOPG), is a good candidate for most cases.^2^ There are exceptions where carbon-based electrodes are not ideal, particularly in cases where the reaction demands a highly oxidative potential, as in the oxygen evolution reaction (OER). At typical OER potentials carbon can undergo oxidation and result in misleading interpretation of the current unless the products are measured. To address this challenge, it is recommended to opt for an alternative material like gold. However, it is important to note that even Au electrodes can develop a passivating gold-oxide layer under certain conditions.^3^

Glassy carbon is a non-porous, inert material that is electrically conductive and can be polished to a very high degree of flatness, usually used with a mirror-polished surface. HOPG is a form of graphite that consists of stacked layers of graphene with carbon atoms arranged in a hexagonal lattice. This makes HOPG a highly flat and conductive material.

Both glassy carbon and HOPG have been shown to be effective support materials for a wide variety of nanoparticles and small clusters.^2,4^ They are also relatively inexpensive and easy to obtain. While HOPG tends to be more brittle and is available in fewer shapes, its exceptionally high flatness and cleanliness make it an ideal support for working with small entities that can be characterized by scanning tunneling microscopy (STM). On the other hand, glassy carbon, while not as well-defined on its surface, offers versatility in electrode shapes and a high level of purity, making it a favorable support option when working with nanoparticles, especially in cases where STM characterization is not a requirement.

After selecting the support, the next step involves determining the optimal cleaning procedure to prepare it for nanoparticle deposition. In this discussion, we will explore the specific scenario of working with glassy carbon, given its extensive use as a support material in our research. We suggest initiating the process with physical polishing, utilizing an 8-shaped movement on a microcloth disc, starting with a coarser grain of ¼ μm and progressing to the finest grain (like 0.04 μm) to achieve the flattest surface.^5^ Depending on the reaction of interest, the composition of the polishing paste or suspension must vary to prevent significant contamination from influencing the reaction, particularly in the case of CO_2_ reduction, where alumina paste is advised against to avoid an increased in the parasitic HER activity.^6^ Following physical polishing, we recommend a chemical acid treatment to eliminate any remaining traces of metal and create nanometrical variations in electrode roughness.

**Fig. 3:**
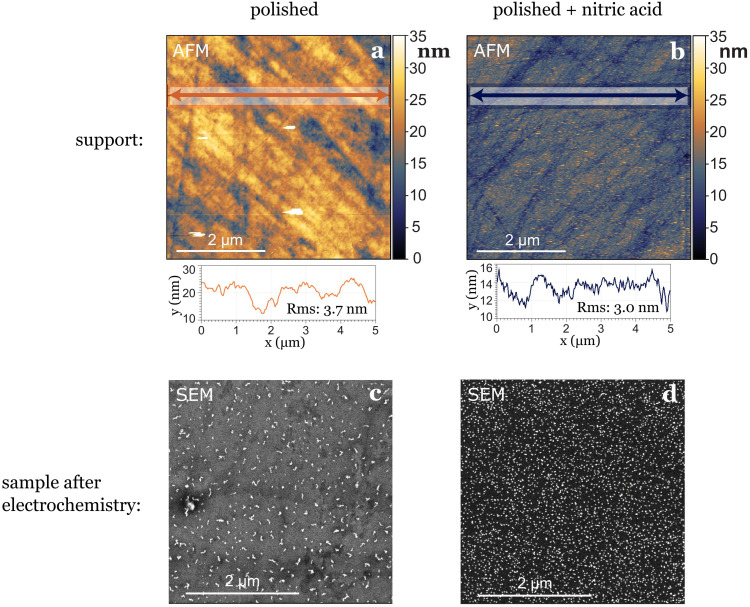
The pre-treatment of the support can influence the stabilization of nanoparticles. An example is shown here for two different samples where only the non-UHV part of the treatment of the support was changed. (a) Roughness of glassy carbon surface after cleaning it by polishing only, before deposition of nanoparticles, (b) roughness of glassy carbon surface directly after cleaning by polishing and nitric acid treatment, before deposition of nanoparticles. Line scans in (a) and (b) show that the root mean square (RMS) roughness is higher for the surface not treated with nitric acid. The same coverage of 5 nm Au nanoparticles were deposited on both supports. Panels (c) and (d) show SEM images of the samples after undergoing the same electrochemical test, depicting a difference in the agglomeration of the nanoparticles.

In our study, where glassy carbon served as the support, we investigated three different cleaning procedures: the first involved solely physical polishing of the electrode, the second consisted on immersing the electrode in aqua regia for one hour after polishing, and the third on immersing the electrode in concentrated nitric acid for the same duration after polishing. Following each chemical treatment, all electrodes underwent sonication in water and ethanol at least three times before use. Utilizing atomic force microscopy (AFM) prior to nanoparticle deposition revealed differences in electrode roughness: freshly polished samples exhibited greater roughness variations, nitric acid-treated samples displayed smaller in height but more evenly distributed roughness (Fig. 3), while aqua regia-treated samples fell in between. After electrochemical testing, we observed the agglomeration and dissolution of gold nanoparticles on the electrode surfaces with scanning electron microscopy (SEM). The nanoparticles deposited on the glassy carbon treated with nitric acid remained more separated and uniformly distributed, while the ones deposited on the just polished support agglomerated into bigger string-like structures. This leads to the conclusion that the small (1–4 nm high) and evenly distributed roughness induced by nitric acid treatment played a stabilizing role for the nanoparticles (Fig. 3). However, generally, drawing conclusions about post-electrochemistry agglomeration of nanoparticles on the electrode's surface is challenging due to a lack of reproducibility in the procedure, as the extraction results in the loss of potential control.^7^

As general advice when starting to work with a new electrocatalytic support, we recommend spending time optimizing its preparation and having several reference measurements on blank supports.

## Cleaning methods in UHV

### Cleaning methods

When the nanoparticles are synthesized and deposited onto the substrates under ultrahigh vacuum (UHV) conditions, additional steps can be taken to clean the surface of the support in UHV before adding the nanoparticles. Typical surface science cleaning methods in UHV include sputtering the sample surface with Ar^+^ ions to remove the top layers of atoms, and/or annealing at an elevated temperature to remove contaminants from the air exposure such as carbon residues. For these methods, it is important to understand the influence of the treatment on both the composition and structure of the surface.

### Characterization methods

Once the substrate has been cleaned, several microscopy and spectroscopy methods can be used to study the cleanliness and topography of the surface. To investigate the structure of the surface, electron microscopy and scanning probe microscopy techniques may be used, which depending on the specific technique chosen provides different types of information about the surface such as local topography or physical contaminants. To investigate the presence of trace amounts of, *e.g.*, foreign metals in the carbon surface, one can also use X-ray photoelectron spectroscopy (XPS) or ion scattering spectroscopy (ISS), also known as low-energy ion scattering (LEIS). The latter is the more surface sensitive technique, as most of the signal detected arises due to light ions elastically scattered by the outermost atoms of the sample surface, and there is a high cross-section for neutralization of the ions below the first atomic layer in the surface. For instance, we have shown that it is an order of magnitude more sensitive to Pt than XPS.^8^ Since it is highly sensitive to small amounts of material, ISS can be used to check the cleanliness of the support before nanoparticles are deposited, for example to check that any previous catalyst traces are fully removed if the support has been re-used between tests. While highly sensitive, these techniques usually probe a given fraction of the sample surface (for example 1 mm^2^), which means that, *e.g.*, the edges of the support may be unprobed.

## Intrinsic support contamination

Even by following a thorough cleaning procedure outside and inside the deposition chamber, the cleanliness of the support is not guaranteed.

**Fig. 4:**
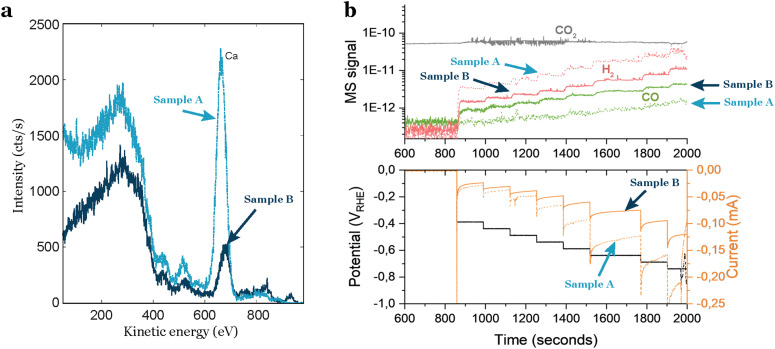
Impact of impurities in the support on the electrocatalytic activity of a sample. (a) ISS spectra of two glassy carbon supports from the same batch, but with different amounts of impurities. (b) Electrochemical performance of 3.5 nm Au nanoparticles with 5% coverage of the electrode. The sample contaminated by the support exhibits increased H_2_ production, overshadowing the CO_2_R activity.

Researchers often assume that commercially acquired products are consistently the same, but this is a misconception. Material providers typically do not verify all the physical properties of each product they sell. Consequently, when obtaining electrochemical supports, caution is required due to the potential intrinsic contaminations in each individual support. Based on our experience, intrinsic contamination persists even after polishing or chemical treatment, as it likely exists in several or all layers of the support. When dealing with industrially-made materials, researchers should know the manufacturing process of the product. This investigation is crucial for identifying potential sources of physical variations and uncertainties related to the presence of contaminants in the material. In our experience with glassy carbons, we observed significant variations in intrinsic contamination based on the provider, batch, and even individual supports from the same batch. To gain insights into the average amount and type of contamination in glassy carbons, researchers can inquire about the ash content, which refers to the residual inorganic material left behind after the carbonaceous components are burned away. This measure provides valuable information about potential impurities and the overall cleanliness of the glassy carbon support, although incomplete since elements like sulfur may not be detected. In Fig. 4(a) this difference is noticeable: two glassy carbons from the same batch, treated with the same polishing and cleaning procedure, exhibit distinct different contamination levels, as indicated by the ISS results. One glassy carbon shows a higher intensity in the background and a more pronounced peak attributed to calcium. These variations in support contamination directly influence sample performance, as illustrated in Fig. 4(b), where the glassy carbon with impurities generates a significantly larger amount of H_2_. This results in a higher current response (Fig. 4(b) bottom) and diminishes the CO_2_ reduction activity.

This intrinsic contamination can be identified by ISS as we did in our case, but also by doing the blank test with each one of the GCs and keeping track of them before the nanoparticle deposition.

## Induced contamination

**Fig. 5:**
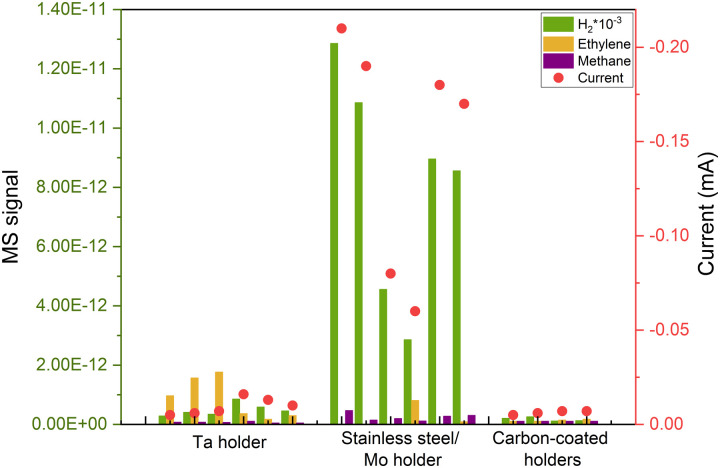
Repetitions of COR of the same deposited sample (5 nm 5% coverage Cu nanoparticles), with different responses due to induced contamination from the UHV cleaning method. The results became reproducible after covering the electrode's holder with carbon. All measurements are done at the same applied potential of −0.75 V_RHE_.

After successfully minimizing contamination from the support and eliminating potential atmospheric contaminants by sputtering in the UHV system, we anticipated a robust, impurity-free system. However, at very cathodic potentials, we observed unequal behaviors in nominally identical samples, despite no apparent distinctions in the ISS or XPS spectra. This is the case illustrated in Fig. 5, where we repeated identical 5 nm Cu nanoparticles with 5% coverage of the electrode and the samples yielded significantly different results at the same electrochemical conditions. The variations included changes in selectivity and current response, predominantly characterized by a noticeable increase in the hydrogen signal, the cause of which was unknown at the time.

Given the capacity of Cu to rapidly oxidize in air, we conducted a study by altering the gas environment that the nanoparticles would be exposed to during the initial 8 hours after being extracted from the cluster source. Surprisingly, the variations in gas did not influence the performance of the nanoparticles, yet the issue of irreproducibility persisted.

**Fig. 6:**
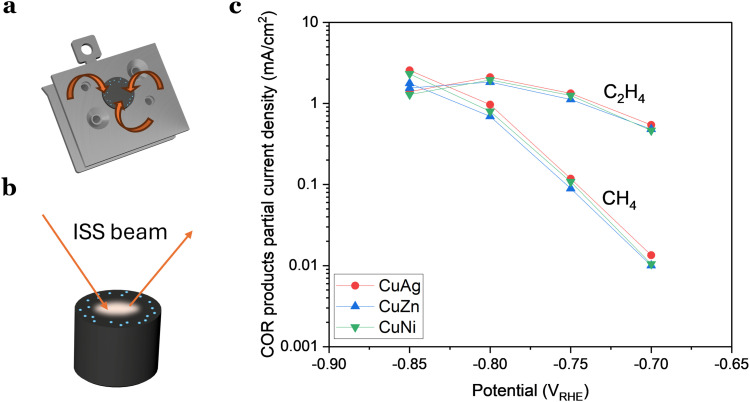
(a) and (b) Schematic representation of how the contamination was induced from the support but overlooked by ISS, emphasizing that the issue was related to the detection location. (c) Partial current densities of ethylene and methane for doped Cu nanoparticles (8 nm, 3% coverage). The addition of 1 at% does not have an impact in the activity or selectivity of the nanoparticles.

After multiple repetitions, we realized that the only consistent factor among samples exhibiting a lower H_2_ signal and, consequently, a diminished current response, was the material of the holder used in the cluster source to secure the electrode. Upon investigation, we found that during the sputter cleaning of the sample (as explained above), some of the material from the holder was being sputtered and redeposited on the electrode's surface, leading to induced contamination from the experimental procedure. That caused irreproducible results during electrochemistry as it is shown in Fig. 5. We attempted to illustrate this phenomenon in Fig. 6(a).

We addressed this issue by covering all the electrode holders with graphite, after which the reproducibility issues disappeared (Fig. 5).

As the contamination was undetectable with ISS, we wanted to determine if the reason was that the level of contaminant was so low that was under the detection level of the technique. This would imply that the quantity of contaminant/dopant was in the ppb region. Alternatively, we considered the possibility that ISS, being a localized technique, might not detect contamination in areas where the ISS beam did not probe (Fig. 6(b)).

Based on our experience, the latter scenario appears more plausible, suggesting that hydrogen-active contamination may be concentrated more significantly along the electrode's rim. This is substantiated by results presented in Fig. 6(c), where we examined three different samples with a 1 at% addition of a dopant metal (Ni, Zn, or Ag). The doped Cu nanoparticles exhibited no significant variations in activity or selectivity, implying that this minute amount of added material had a limited impact on the activity.

These observations have led to several conclusions. Firstly, electrochemical performance emerges as the most sensitive technique for surface characterization. Secondly, it highlights the importance of being vigilant at every step in the experimental protocol that could introduce contamination. Even the cleaning steps can introduce errors if not executed accurately.

## Nanoparticle synthesis

In the quest to determine the intrinsic catalytic activity of nanoparticles, the chosen synthesis method plays a critical role, impacting their overall performance. The predominant approach to control size and shape is ligand-based synthesis, and while widely employed it can introduce substantial challenges that hinder the accurate assessment of the catalyst's activity. Often, the lack of control over the coverage of the electrode complicates access to the electrochemical surface area (ECSA), and the variability on interparticle distance renders results obtained at different coverages not easily comparable.^9^ Moreover, the intricate removal of ligands not only poses difficulties but also raises concerns about possible contamination and compromises reactivity due to ligand-induced reactions.^10^

To avoid these issues and attain a clearer understanding of the catalyst's intrinsic activity, it can be an advantage to produce nanoparticles using ligand-free physical gas-phase depositions of nanoparticles with UHV-based equipment.^11^ Among the various methods within this category, cluster sources^12^ can produce nanoparticles of single or mixed compositions using, *e.g.*, magnetron-sputtering of metallic sputter targets. These are usually combined with a mass selector such as a quadrupole or time-of-flight^13^ mass filter, the latter of which can allow for size selection with a mass resolution of *M*/Δ*M* ∼ 20.^12^

**Fig. 7:**
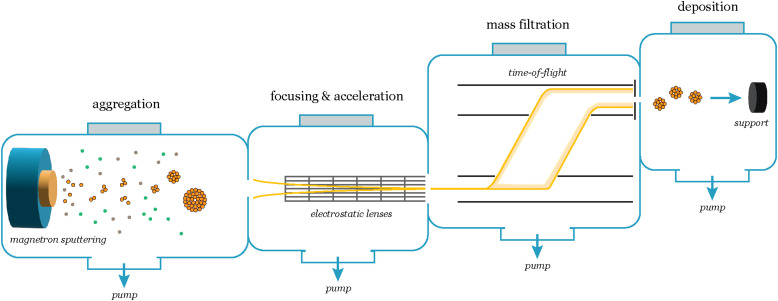
Schematic of the nanoparticle synthesis and mass selection process in a cluster source system. The cluster formation begins in the aggregation zone, where a range of cluster sizes form. A beam of charged particles is then extracted from the aggregation zone and focused and accelerated using an array of electrostatic lenses towards the mass selection chamber, which consists of a lateral time-of-flight mass filter. With the mass filter, the desired mass/charge ratio is selected, and subsequently the particles are deposited onto the support.

In a cluster source system, nanoparticles are first formed by magnetron sputtering in an aggregation zone, then guided as a beam using electrostatic lenses and mass selected before deposition onto the substrate (Fig. 7). The precise number of nanoparticles deposited on the substrates, and thus loading, can be detected from the neutralization current in the same stage, which arises from the charged nanoparticle impinging onto the sample surface. By modelling the nanoparticles with a spherical approximation, the total charge deposited during a deposition can then be used as an estimate of the achieved nanoparticle coverage and ECSA, in lieu of other more precise methods to measure the ECSA such as CO-stripping for precious metals.^14^ A homogeneous distribution of nanoparticles can be achieved across the sample surface by rastering, either the nanoparticle beam itself using a rastering lens, or by rastering the sample in front of the beam.^15^ This technique has the advantage of creating very well-controlled samples with very low loadings, where each nanoparticle and the nanoparticle distribution across the surface are generally well-defined. It is very suitable for model studies of nanoparticle structures and should be coupled with highly sensitive UHV-based or electrochemical techniques to evaluate the catalytic properties of the nanoparticles. Limitations of the technique are making samples with higher loadings (>μg cm^−2^) of nanoparticles, and that some non-metallic targets are more difficult to sputter and therefore unsuitable for the cluster source method.

**Fig. 8:**
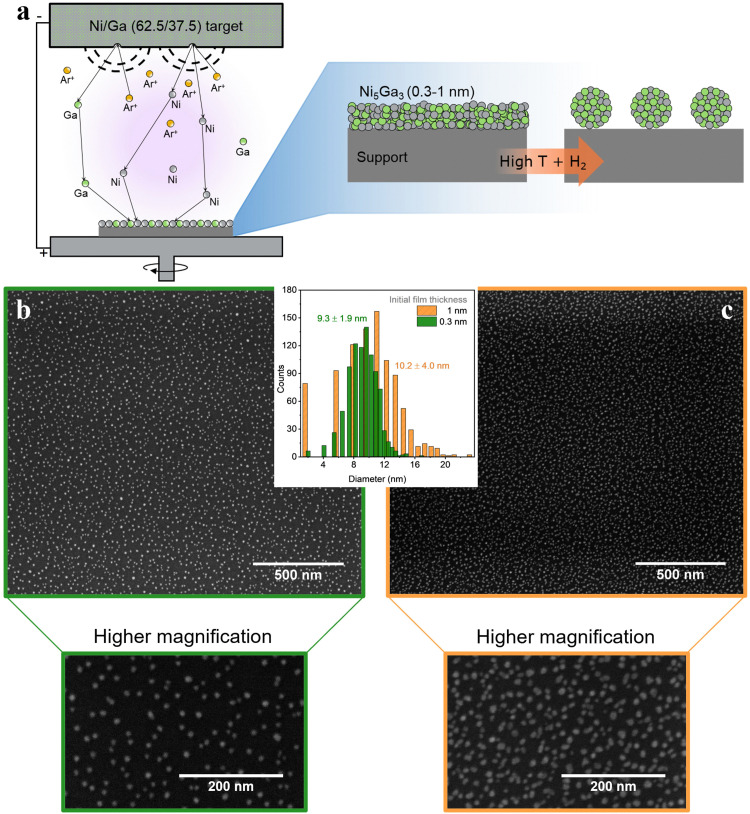
(a) Nanoparticle synthesis schematics, combining magnetron sputtering and annealing; (b) and (c) Ni_5_Ga_3_ thin films of 0.3 (green) and 1 nm (orange), respectively, on an SiO_2_ (50 nm)/Si (350 μm) support annealed at 750 °C for 30 min in 10% H_2_ in He. SEM images were acquired at 5 kV and 0.1 nA.

Another viable strategy is a simple, cost effective, yet effective route: thermal annealing of sputtered metallic thin films. This process is schematically represented in Fig. 8(a). Magnetron sputtering offers the possibility to deposit in a controlled way single metals (and alloys) onto practically any type of support (*e.g.*, SiO_2_ for thermal catalysis, and glassy carbons for electrochemical applications). In simple terms, a metallic target is sputtered by bombardment of Ar^+^ ions. The metallic target acts as the cathode and the sample holder (*i.e.*, the substrate to be deposited) as an anode.

After physical deposition, metallic films can be thermally treated under strict controlled conditions to promote de-wetting of the nanometric films. As result, metal films are converted to discrete metal nanoparticles.^16,17^

Fig. 8(b) and (c) show metallic Ni_5_Ga_3_ nanoparticles formed after thermal annealing of sputtered 0.3 nm and 1 nm films, respectively. The annealing was performed for 30 minutes at 750 °C in 10% H_2_ diluted in He. In simple terms, the difference in surface energy at the interface between the metallic films and the flat substrate leads to the formation of small particle islands, known as de-wetting phenomenon. The procedure yields small and well dispersed nanoparticles with a relatively narrow size distribution, especially in the case of very thin films of 0.3 nm. The obtained high surface area coverage (around 10% for the 0.3 nm films and 20% for the 1 nm one) corresponding to high loading of metal particles while maintaining a relatively narrow size distribution is difficult to obtain with other physical deposition methods since, for example, the stochastic deposition of nanoparticles on the substrate in cluster sources would lead to overlapping nanoparticles, effectively losing the benefit of size selection.^14^

Metallic de-wetting is a complex process, and the optimization of key parameters like annealing temperature and time, nature of the support, inert or reducing atmosphere and metal thickness leads to a precise control of the mean particle size and their distribution.^18,19^ As a example, higher temperatures promote the kinetics of the process whereas thinner metallic films result in the formation of smaller particles. Nevertheless, high temperatures can also lead to pronounced sintering, mainly due to migration and coalescence of smaller particles that become mobile onto the surface. Similarly, metal–support systems of high interaction can lead to metal migration into the bulk. This leads to smaller particles but also metal loss and possible formation of unwanted phases.^20,21^ Moreover, different cooperative effects can be present for multimetallic films, leading to formation of alloyed and/or segregated phases.

Physical deposition and thermal annealing of sputtered thin films holds significant potential as a strategy for creating supported nanoparticles. Nevertheless, the final structure is highly dependent on both the metal phase and support. Annealing conditions should be chosen carefully, depending on the metals of interest, but also on the substrate on which they are to be deposited, which should be stable at the selected conditions. Therefore, each particular system requires a delicate selection of the deposition and annealing conditions (*e.g.*, gas composition, pressure, temperature, and gas flow), to promote the formation of desired size-selected metal nanoparticles.

We acknowledge that the colloidal synthesis of metal nanoparticles following wet-chemistry approaches is widely employed since it is easily accessible without requiring expensive equipment. However, to avoid or minimize any potential contamination sources, the effective removal of capping agents is mandatory without compromising the size, shape, or chemical state of the deposited nanoparticles. Some strategies involve controlled thermal treatments under oxidizing and/or reducing atmospheres,^22^ UV light,^23^ washing treatments with specific solvents,^24^ and plasma treatments.^25,26^ A thorough characterization after treatment is required to know if significant changes occurred during the process.

## Confirming the homogeneity of the dispersion of nanoparticles

The nanoparticles density can influence their reactivity by altering their electrochemical surroundings (*e.g.*, pH variations, diffusion layer superposition).^1,27^ Therefore, achieving a uniform distribution of these small catalysts, while preserving a large interparticle distance, is crucial for obtaining an averaged activity similar to that of isolated nanoparticles. To assess sample homogeneity, we recommend utilizing nanometric resolution techniques like SEM or AFM. These methods provide an overview of the sample and sufficient resolution to identify individual nanoparticles down to 2.5 nm in size on the same support used for electrochemical tests.

**Fig. 9:**
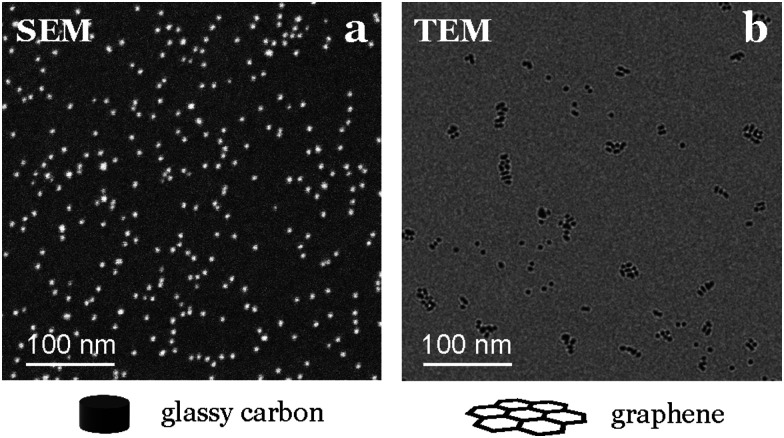
Differences in nanoparticle distribution depending on the support. (a) SEM image of 5% coverage of 3.5 nm Au nanoparticles on glassy carbon. (b) TEM image at the same scale with 2% coverage of Au nanoparticles on graphene.

We emphasize the importance of using a technique directly applicable to the support used in activity tests, since we have observed significant variations in nanoparticle dispersion and homogeneity for the same composition and deposition conditions when changing the support material. For instance, when depositing nanoparticles onto graphene TEM grids, they appear to align to the defects in the graphene structure, a phenomenon not observed on the glassy carbon used for testing (see Fig. 9).

## Electrochemical techniques and product detection

**Fig. 10:**
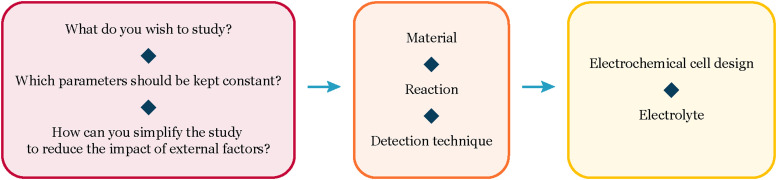
Decision map – electrochemical project definition.

Assessing the electrochemical performance of the samples is not straightforward either, as the goal is to evaluate the electrochemical activity and selectivity of extremely small loadings of nanoparticles (Fig. 10).

The first consideration involves the cell design and the analytical technique chosen for product detection. Given the very low total quantity of products expected, an extremely sensitive technique becomes essential.

### Product detection

There are several strategies to detect tiny amounts of product, with one of the most cost-effective methods being the rotating ring disk electrode (RRDE). This purely electrochemical technique involves using a rotating electrode system, typically consisting of a center disk electrode and a surrounding ring electrode. The center disk electrode is responsible for the electrochemical reaction under study, while the ring electrode is employed for product detection. Through the rotation of the electrode assembly, the products generated at the disk electrode are transported to the ring electrode for detection, involving the reverse reaction compared to that occurring at the disk electrode. By measuring the current generated in the ring, we can quantify the amount of product that reacted as it formed on the working electrode.^28,29^ RRDE is commonly used to detect products like H_2_O_2_ in the case of oxygen reduction reaction (ORR) or CO in the case of CO_2_ reduction (CO_2_R).^28,30^ This technique can be highly sensitive and relatively easy to implement, but it is restricted to the detection of a single product. In most cases, this limitation prevents obtaining a thorough overview of the studied reaction.

To detect and quantify multiple products, an analytical technique is needed. The most commonly employed methods for product detection in electrochemistry are gas chromatography coupled with specific detectors for gas products and high-performance liquid chromatography (HPLC) for liquid products. Nevertheless, these techniques often exhibit a relatively high detection limit, making them unsuitable when working with low amounts of catalyst.

Mass spectrometry can offer the required sensitivity and allows the detection of most products, even with online product detection. In cases where the products have very similar masses, contemplating a separation technique before introducing the sample into the mass spectrometer is advisable, for example, gas chromatography.^31^ Another parameter to factor in when working with mass spectrometry is the ionization energy of the filament, as the fragmentation of molecules can vary depending on this energy.^32^ For instance, in the case of CO_2_R, a low ionization energy (*e.g.*, 22 eV) is necessary to prevent the fragmentation of CO_2_ into CO, thereby avoiding the shadowing of the produced CO. We advise finding an optimal ionization energy depending on the products that one wants to measure to get a maximized sensitivity and the separation between their peaks.

Connecting a mass spectrometer to an electrochemical cell is challenging due to the significant pressure difference, with the cell at 1 bar and the mass spectrometer at 10^−9^ bar. Different strategies, such as using a membrane with differential pumping (DEMS,^33^ OLEMS^34,35^), a perforated chip that a limits the flow of molecules,^36^ or an orifice,^37^ can be employed to address this issue. Depending on the specific connection between the electrochemical cell and the mass-spectrometer, we are typically constrained to a specific cell design, which should be chosen carefully based on the expected reaction conditions.

### Cell design

To draw conclusions about a catalyst's intrinsic properties, it is necessary to maintain a consistent catalyst environment across the sample. Classical flow cells are not advised for fundamental studies because the catalyst's surroundings can change based on its placement within the cell, influenced by convection and pH variations during the reaction. When dealing with low current densities, it is better to use a stagnant cell to minimize damage to the sample and reduce external factors that could impact the measured activity.

In many cases and reactions, it is necessary not to be limited by mass transport in order to assess the intrinsic activity of the catalyst. By enhancing the diffusion using a rotating disk electrode setup it is possible to mitigate the effects of mass-transport limitations. However, it is essential to be cautious about maintaining consistent convective conditions, as these play a vital role in ensuring accurate comparisons between experiments, with changes in diffusion directly influencing reaction selectivity.^28^

### Electrochemical techniques

**Fig. 11:**
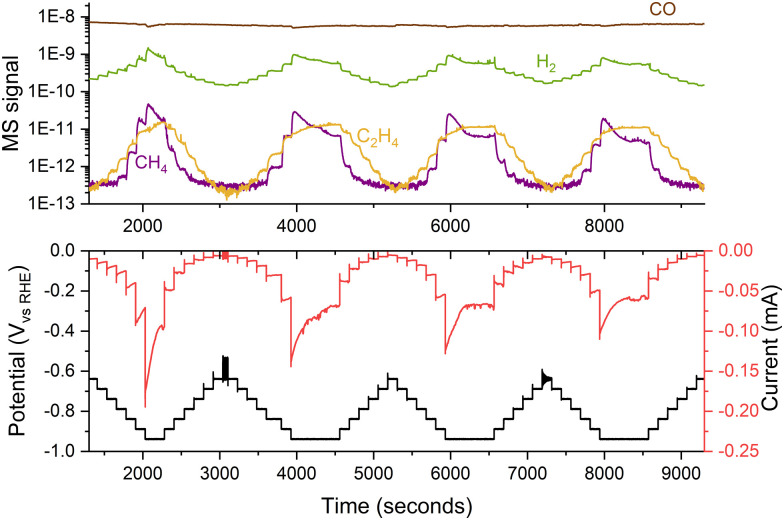
Variations in the performance of 5 nm 5% coverage Cu nanoparticles for COR. The experiment consisted of several consecutive potential steps of 50 mV, measuring the current response and the generated products with a mass spectrometer. The experiment was performed in a stagnant thin-layer electrochemical cell interfaced to the mass spectrometer through a microporous aqueous chip.^32^

When working with nanoparticles in electrochemistry, it is essential to handle them with care as they can easily dissolve and sinter under harsh conditions. To minimize this effect, samples should be kept under potential control at all times, from their introduction in the electrochemical cell, ensuring that the catalyst enters the electrolyte at a potential where it is stable.^38^ Additionally, one should avoid potentials that might cause nanoparticles to corrode as ions into the electrolyte. Operating within a narrow potential range is advisable to prevent damage to the sample. Using small potential steps (*e.g.*, 50 mV) can reduce changes in the sample by enabling precise control and modulation of electrochemical processes,^39,40^ even though variations in performance may still be observed during medium-term experiments, as depicted in Fig. 11.

## Conclusion

Working with small quantities of catalyst requires mitigating the influence of external factors, such as contamination or modifications in the physical properties of the support, for a precise evaluation of the catalyst's intrinsic activity. In this perspective, we aim to equip researchers with the essential tools for effectively working with well-defined, physically deposited nanoparticles. This enables a more refined exploration of the impact of altering key physical parameters, such as size, shape, or composition, on the catalytic behavior of nanoparticles.

The insights shared in this perspective serve as a roadmap for future research endeavors in nanoparticle electrochemistry. By providing a comprehensive guide that spans from support preparation, including considerations for intrinsic and induced contaminations, to nanoparticles synthesis focusing on avoiding chemical contamination and controlling key factors like the exact ECSA, we aim to empower researchers on how to effectively work with such systems in electrochemistry. As the field advances, embracing the intricacies of working with nanoparticles becomes fundamental. Future studies can build upon this foundation, expanding the scope of nanoparticle research and uncovering new dimensions of their catalytic activity.

## Data availability

Data for this article, including the EC-MS data used in Fig. 4–6 and 11 will be available at DTU data at https://data.dtu.dk/.

## Conflicts of interest

There are no conflicts to declare.
